# Underwater Single-Photon 3D Reconstruction Algorithm Based on K-Nearest Neighbor

**DOI:** 10.3390/s24134401

**Published:** 2024-07-07

**Authors:** Hui Wang, Su Qiu, Taoran Lu, Yanjin Kuang, Weiqi Jin

**Affiliations:** MOE Key Laboratory of Photoelectronic Imaging Technology and System, Beijing Institute of Technology, Beijing 100081, China; 3120210606@bit.edu.cn (H.W.); 3120210536@bit.edu.cn (T.L.); 3220220524@bit.edu.cn (Y.K.); jinwq@bit.edu.cn (W.J.)

**Keywords:** single-photon imaging, 3D reconstruction, K-nearest neighbor algorithm, SPAD, underwater imaging

## Abstract

The high sensitivity and picosecond time resolution of single-photon avalanche diodes (SPADs) can improve the operational range and imaging accuracy of underwater detection systems. When an underwater SPAD imaging system is used to detect targets, backward-scattering caused by particles in water often results in the poor quality of the reconstructed underwater image. Although methods such as simple pixel accumulation have been proven to be effective for time–photon histogram reconstruction, they perform unsatisfactorily in a highly scattering environment. Therefore, new reconstruction methods are necessary for underwater SPAD detection to obtain high-resolution images. In this paper, we propose an algorithm that reconstructs high-resolution depth profiles of underwater targets from a time–photon histogram by employing the K-nearest neighbor (KNN) to classify multiple targets and the background. The results contribute to the performance of pixel accumulation and depth estimation algorithms such as pixel cross-correlation and ManiPoP. We use public experimental data sets and underwater simulation data to verify the effectiveness of the proposed algorithm. The results of our algorithm show that the root mean square errors (RMSEs) of land targets and simulated underwater targets are reduced by 57.12% and 23.45%, respectively, achieving high-resolution single-photon depth profile reconstruction.

## 1. Introduction

Single-photon imaging can effectively improve imaging resolution and the range of detection systems, and it has been widely applied in fields such as underwater target detection [1,2], fluorescence lifetime imaging [3,4], and medical image diagnostics [5]. Single-photon avalanche diodes (SPADs) are able to detect single photons via an avalanche process [6]. Moreover, picosecond time resolution can be achieved by integrating time-correlated single-photon counting (TCSPC) [7] or time gating (TG) [8,9,10,11], which allows for time stamps of the photons arriving, thereby achieving three-dimensional imaging. However, the dead time and timing jitter of SPADs, typically ranging from hundreds of picoseconds to nanoseconds, hinder the time resolution.

When applying imaging to an underwater environment, the greatest challenge comes from the absorption of light through water and the scattering of light via particles [12]. The high sensitivity of SPADs to photons allows for the detection of photons despite this absorption, but this process still suffers from scattering [13], especially in turbid water, which leads to a significant decrease in the signal-to-noise ratio (SNR) of the detection results. Therefore, it is necessary to deploy single-photon image reconstruction algorithms to perform noise reduction on a time–photon histogram to achieve high-resolution single-photon depth profiles.

**Figure 1:**
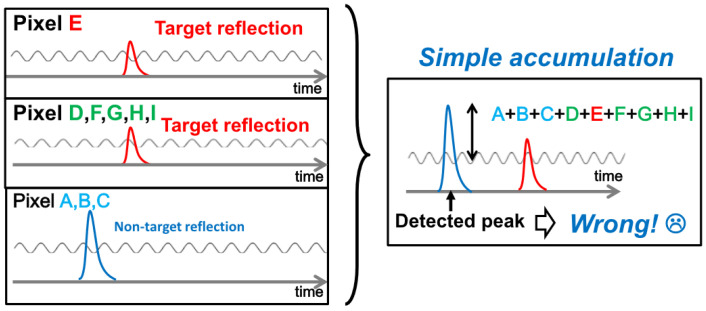
Simple pixel accumulation depth estimation error.

Compared to working with individual pixels, illuminating all pixels in the field of view with a laser simultaneously reduces the maximum illumination of each pixel and also decreases the SNR of the detection results [14]. In order to improve the SNR, pixel accumulation is performed during the LiDAR data collection process, utilizing digital signal filtering and multi-pulse accumulation to enhance the SNR of the echo signal. Traditional LiDAR employs two types of pixel accumulation [15]: accumulating multiple measurements within the same pixel, known as time averaging, and accumulating the results of adjacent pixels, known as spatial averaging. Both accumulation methods improve the signal-to-background ratio (SBR) with the square root of accumulated samples. However, the time-averaging method requires a trade-off with FPS. In order to improve the SBR of LiDAR without reducing the FPS [16], spatial accumulation, or simple accumulation [17], is adopted. This method is effective only when all of the pixels being accumulated detect the same target. The accumulation of non-target pixels may lead to significant depth estimation errors in the final reconstruction result (as shown in Figure 1).

Through the simple pixel accumulation algorithm, it is possible to remove the influence of noise to some extent, thereby obtaining the accurate peak position of the photon signal in the histogram. However, the simple pixel accumulation method will blur the boundaries of the target, leading to a decrease in the quality of the reconstructed depth profiles, and it may result in incorrect depth estimation for targets. Therefore, it is necessary to classify targets, noise, and the background before pixel accumulation. As a multi-classifier, the K-nearest neighbor (KNN) algorithm can satisfy the classification of multiple targets, backgrounds, and noise with good prediction performance and insensitivity to outliers. As a typical example of ”lazy learning”, the training cost of KNN is very low. Therefore, we adopted KNN combined with the pixel accumulation algorithm to perform noise reduction with a histogram and simultaneously use classic single-photon depth profile reconstruction algorithms such as cross-correlation [18,19] and ManiPoP [20] to reconstruct depth profiles with millimeter-level depth uncertainty from the time–photon histogram, achieving high-precision three-dimensional reconstruction of an underwater target.

## 2. SPAD Underwater Optical Imaging Experimental System

**Figure 2:**
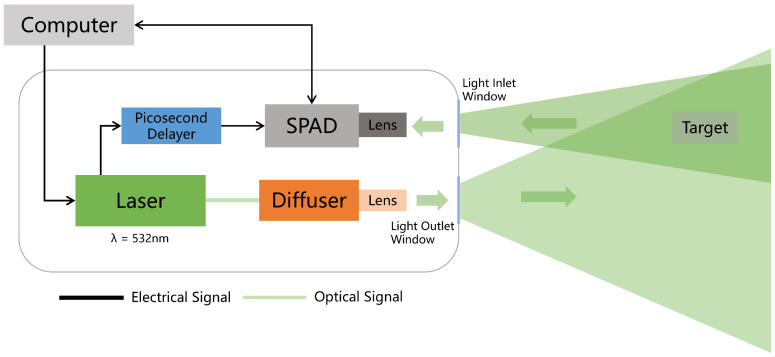
SPAD underwater optical imaging experimental system schematic diagram.

The underwater SPAD imaging system is shown in Figure 2. This system is controlled via a computer. The laser emits a laser beam, which is focused through a lens to illuminate the target at a certain distance. Simultaneously with the emission of the laser, a synchronized trigger signal is generated, which is calibrated via the picosecond delayer and then transmitted to the SPAD camera. Then, the SPAD camera is ready for the detection of the photons returned from the target.

In this system, a time-to-digital converter (TDC) is integrated into a SPAD for underwater target detection based on TCSPC. Integrating TDC into the chip causes a decrease in the pixel fill factor. The chosen SPAD detector is the PF32 SPAD produced by Photon Force. The PF32 SPAD array, while integrating a TDC, uses a microscope array to increase the effective fill factor of the single-photon camera to 20%. The TDC has a time resolution of 10 bits, with a bin width of 55 ps, providing very high precision for photon time detection. Furthermore, when the PF32 SPAD camera measures the time of the returned photons, it uses a reverse start–stop mode, which means that, when a single photon is detected, the TDC starts timing, and the next laser-synchronized trigger signal stops the TDC timing. This mode significantly reduces the power consumption of the SPAD camera.

**Figure 3:**
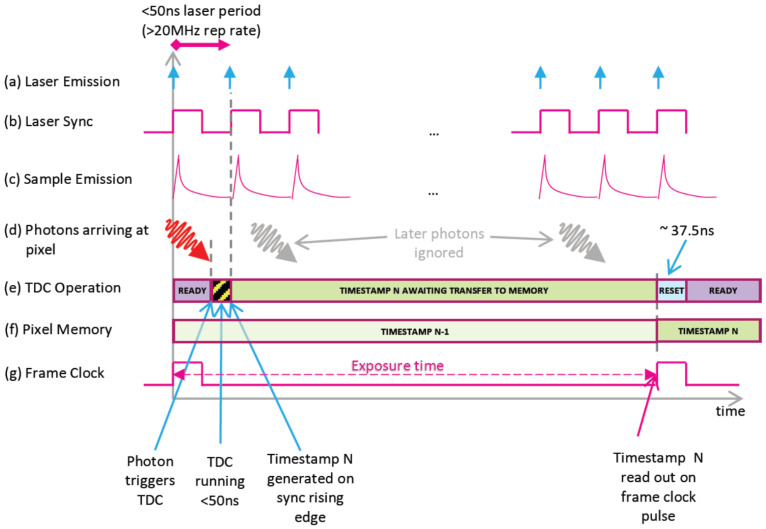
Schematic diagram of PF32 SPAD photon time measurement.

The specific method of photon time measurement based on the TDC reverse start–stop mode is shown in Figure 3. The pulse laser repetition frequency is set to 20 MHz, and the laser beam is emitted onto the target sample. Simultaneously with the emission of the laser beam, the synchronized trigger signal from the laser is transmitted to the PF32 SPAD. The TDC of each pixel is in a ready state until a returned photon is detected, at which point the TDC starts timing and stops timing upon receiving the next laser-synchronized pulse signal. The recorded photon timestamp waits to be transmitted to memory, during which the TDC cannot record any photon information, meaning that each TDC can only generate one photon timestamp per frame. By subtracting the start and stop timing moments, the flight time measurement value $T_{0}$ is obtained. The actual photon flight time is $T_{l}$ = $T_{r}$ − $T_{0}$, where $T_{r}$ is the laser pulse period. After multiple pulse measurements, the histogram information needs to be converted in order to obtain the real photon flight time.

In the underwater SPAD imaging system, although the high sensitivity of SPAD allows low laser power, it still strictly requires parameters such as the laser repetition frequency, pulse width, and central wavelength. When planning to observe underwater targets at a distance of 15 m, the selected light source was the PicoQuant VisUV-532 picosecond pulse laser produced by PicoQuant. Moreover, according to the target distance, the laser repetition rate should be set to 10 MHz. The VisUV-532 obtains 532-nm picosecond pulses based on a fiber amplifier frequency conversion from a main oscillator fiber amplifier. It can be applied to laser radar measurement/ranging/satellite remote sensing measurement, fluorescence lifetime imaging, time-resolved fluorescence imaging, and so on. The main parameters of the imaging system are shown in Table 1.

Additionally, it is crucial to minimize the timing synchronization delay between the laser and the SPAD. Therefore, we utilized a picosecond delayer (PSD-MOD) produced by Micro Photon Devices to calibrate the synchronization trigger signal generated via the laser, which ensures the stability of the synchronization signal jitter between the laser and the SPAD. In addition to adjusting the delay of the synchronization trigger signal, the PSO-MOD picosecond delayer can also adjust the amplitude of the pulse signal. The PF32 SPAD camera requires the synchronization trigger signal voltage to be 3.3 V. The laser synchronous trigger signal adjusted via the picosecond delayer can perfectly adapt to the requirements of the PF32 SPAD camera and improve the signal stability of the system.

## 3. Single-Photon 3D Reconstruction Algorithm Based on KNN

### 3.1. The Principle of the KNN Algorithm

KNN is a classification and regression method that can efficiently solve prediction and classification problems with specialized datasets. It works by determining the K-nearest neighbors to each test point based on a set of predefined decision rules in order to select the corresponding class from the K neighbors. The selected class is then assigned to the test point.

The steps of KNN are mainly as follows:

(1) Calculate the distance between each data point in the test set and every sample point in the training set;
(2) Sort the obtained distances in ascending order;
(3) Select the first K training samples that are closest to the test point and count the frequency of each class among these K neighbors;
(4) Determine the class of the test point based on the decision rule.

In the steps of KNN, the primary factors determining the final effect of the algorithm are the distance measurement scheme, the K value, and the classification decision rule. The significance of the distance between two sample points in KNN reflects the similarity of the features of these two sample points. Currently, there are various distance measurement methods available for selection, with commonly used methods including Euclidean distance, Manhattan distance, and Chebyshev distance. In this paper, Euclidean distance was chosen as the measure of feature similarity. The Euclidean distance between points *A* and *B* in n-dimensional space is given by Equation  (1)

(1) $$d\left(A,B\right)=\sqrt{\sum_{i=1}^{n}{\left(x_{ia}-x_{ib}\right)}^{2}}$$

The selection of the K value is also crucial to the results of the algorithm. The K value in KNN refers to the number of neighbor samples to consider when predicting the class of a sample to be classified. A smaller K value means that the model becomes more complex and is more likely to be influenced by noise or outlier data in the training set, potentially leading to overfitting. Conversely, a larger K value simplifies the model, which may lead to underfitting, as the classifier may incorrectly classify test samples by including distant, non-similar samples among the K neighbors. In practice, a small K value is initially chosen, and the optimal K value is determined through methods such as cross-validation.

After determining the K value, based on the selected distance measurement scheme, the K-nearest training sample points to the test sample point are found. The final class prediction result for the test sample point is then obtained according to the classification decision rule. The basic assumption of KNN is the closer, the more similar. The most commonly used classification decision rule is based on the majority rule, where the class label of the test sample is determined according to the most common class label among its K neighbors.

**Figure 4:**
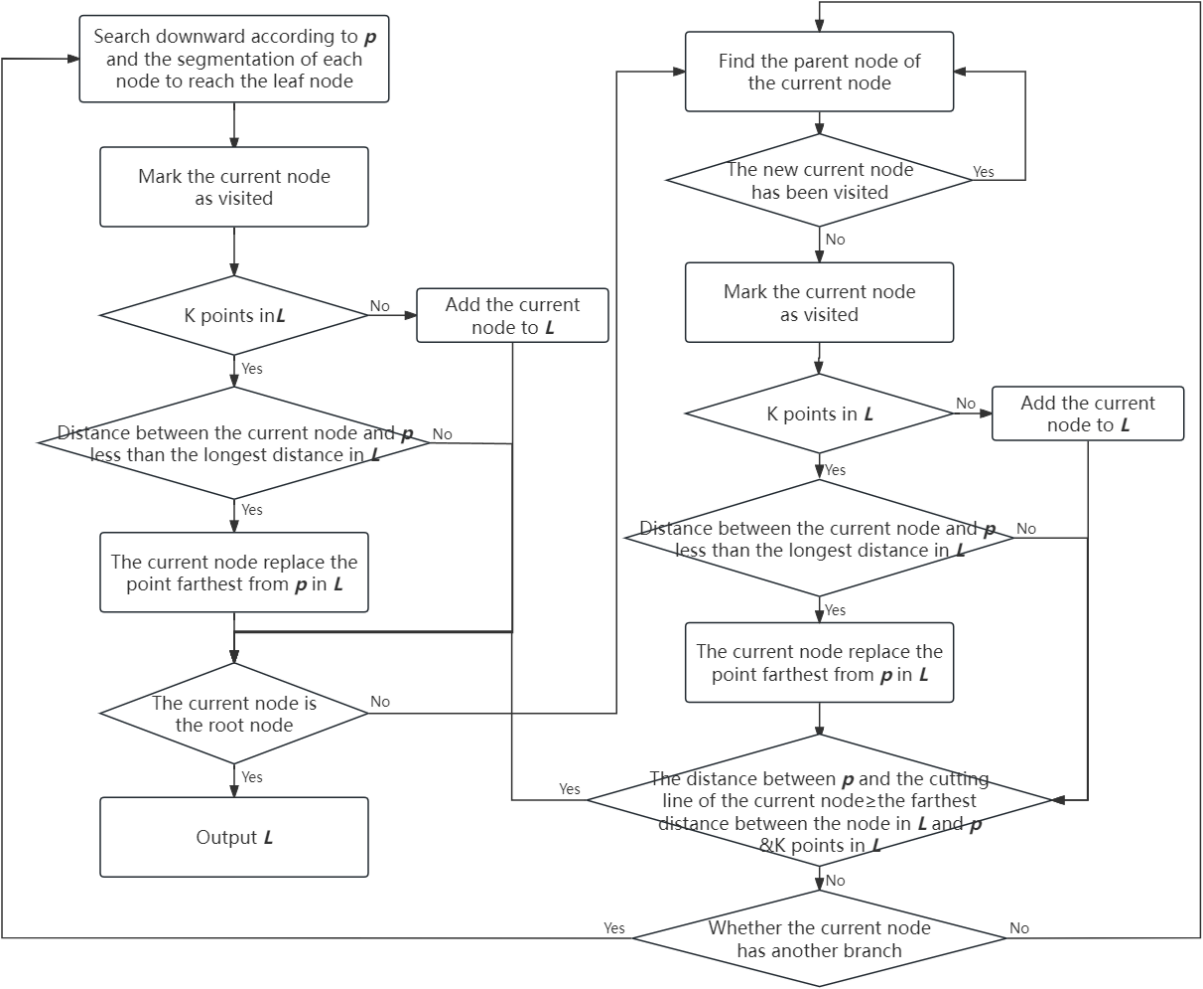
Data search process based on kd-tree.

The training cost of KNN is very low, and the reason for this low training cost is that, during the training stage, KNN merely saves the sample data. If the training set is large, it requires a significant amount of storage space. Additionally, the large amount of data also brings challenges during the testing process. For example, it is necessary to calculate the distance from the test sample to each training sample, which involves all features. When both the amount of data and feature dimensions are large, the computational load of the KNN algorithm becomes substantial and time-consuming. Therefore, in order to enhance the search speed of KNN, a special data storage format, namely a k-Dimensional Tree (kd-tree), is adopted to reduce the number of calculations between the test sample and the training sample. The kd-tree is a binary tree-based data storage format that partitions data points in k-dimensional space, thereby reducing the time cost of data search through efficient indexing. Assuming the test sample point is ***p***, and ***L*** is a list that saves the K training sample points closest to ***p***, the data search process based on the kd-tree is shown in Figure 4.

The KNN algorithm is simple, easy to understand, and very easy to implement, often achieving good predictive results in many cases. However, KNN also has some disadvantages. When the sample data size is too large or the dimensionality of the sample data features is too high, the running time of the algorithm will significantly increase. Furthermore, the predictive performance of KNN is highly dependent on the training set. If certain categories of data in the training set are incorrect, it will directly lead to a decrease in the accuracy of the algorithm.

### 3.2. Principle of Single-Photon 3D Reconstruction Algorithm Based on KNN

After using SPADs for target detection, $N_{r}$ × $N_{c}$ time–photon histograms will be obtained, where each histogram has *h* time bins. The histogram for the pixel coordinates (*i*, *j*) can be expressed as Equation (2):

(2) $$x_{i,j}={\left[x_{i,j}^{1},x_{i,j}^{2},x_{i,j}^{3},\ldots ,x_{i,j}^{h}\right]}^{T},1\leq i\leq N_{r},1\leq j\leq N_{c}$$

The data matrix of the entire SPAD array can be represented as Equation (3):

(3) $$X=\left\{x_{i,j},1\leq i\leq N_{r},1\leq j\leq N_{c}\right\}$$

The KNN algorithm requires calculating the distance between the test sample points and training sample points. It is clearly impractical to use the number of photons saved in each time bin of the time–photon histogram as the feature. The number of time bins is usually in the thousands, making the computational cost prohibitively high. Therefore, four features were selected from the original histogram to calculate the distance between each sample’s four features as the sample similarity. The four features are as follows:

(1) Peak photon number

(4) $$f_{i,j,1}=\max_{1\leq t\leq h}x_{i,j,t}$$

The peak photon number is the photon number of the maximum bin in the time–photon histogram, which stands for the intensity information of the pixel.

(2) Peak position

(5) $$f_{i,j,2}=t\cdot \varphi \left(x_{i,j,t},f_{i,j,1}\right),\varphi \left(u,v\right)=\left\{\begin{array}{c} 1,u=v\\ 0,u\neq v \end{array}\right.$$

The peak position stands for the time position where SPAD detectors received the most photons. If multiple peak positions appear in the histogram, the time bin where the first maximum value is located is taken as the peak position. The depth information of the pixel is calculated from the peak position.

(3) Peak power

(6) $$f_{i,j,3}=\sum_{t=f_{i,j,2}-\Delta t}^{t=f_{i,j,2}+\Delta t}x_{i,j,t},\Delta t=\frac{T_{p}}{2T_{r}}$$

where $T_{p}$ is the laser pulse width and $T_{r}$ is the time resolution of the SPAD camera, that is, the resolution of TDC. The peak power is the total photon number of the time bins near the peak position, which represents the echo signal level.

(4) Total power

(7) $$f_{i,j,4}=\sum_{t=1}^{h}x_{i,j,t}$$

Total power is the average photon number of all time bins in time–photon histogram, and it represents the noise level.

By calculating the four features, the feature vector $f_{i,j}$ of a time–photon histogram can be obtained, as shown in Equation (8):

(8) $$f_{i,j}=\left\{f_{i,j,1},f_{i,j,2},f_{i,j,3},f_{i,j,4}\right\}$$

The successful operation of the KNN-based pixel accumulation algorithm requires obtaining the ground truth of the target histogram. In environments with minimal noise, using a SPAD detector to detect the target at a certain distance results in a time–photon histogram ***Z***, which clearly shows the target features. By processing this histogram through a pixel cross-correlation algorithm, the ground truth of the target depth profile can be obtained. Then, by manually marking the classes of different targets in the ground truth, a labeled map ***Y*** can be obtained, as shown in Equation (9):

(9) $$Y=\left\{y_{i,j},1\leq i\leq N_{r},1\leq j\leq N_{c}\right\}$$

where $y_{i,j}$ represents the class label for each pixel. Additionally, it is also necessary to calculate the feature vector for the ground truth of the target histogram. Once the feature vector for ***Z*** is obtained, the training set ***P*** is composed of the feature vectors of ***Z*** and the class labels for each pixel, as shown in Equation (10):

(10) $$P=\left\{\left(f_{i,j},y_{i,j}\right),1\leq i\leq N_{r},1\leq j\leq N_{c}\right\}$$

The output of the KNN algorithm is the predicted class of the test single-photon sample. Assuming that the test single-photon sample is ***a***, the Euclidean distance between ***a*** and each single-photon data item in the training set ***P*** is calculated. The K-nearest neighbors to ***a*** within the training set ***P*** are selected, and the neighborhood covering these K-data points is denoted as $N_{K}$(***a***). The class with the highest frequency in $N_{K}$(***a***) is the class of the test single-photon data ***a***. The classification decision rule is shown in Equation (11):

(11) $$y_{a}=\underset{c_{i,j}}{\arg\max}\sum_{p_{i,j}\in N_{K}\left(a\right)}\varphi \left(y_{i,j},c_{i,j}\right)$$

where $c_{i,j}$ is the class label of the K single-photon points in $N_{K}$(***a***), and $y_{i,j}$ is the class label of all targets in ***P***.

The specific process of the KNN-based pixel accumulation algorithm is shown in Algorithm 1. After classifying each pixel through the KNN algorithm, the time–photon histograms of pixels with the same class as each pixel are accumulated within the eight-neighborhood region of each pixel to obtain the single-photon data matrix ***Q***. This step avoids the accumulation of histograms that do not belong to the same target, which could lead to incorrect depth estimation and blurred edges for the reconstructed target.
**Algorithm 1** KNN-based pixel accumulation algorithm.**Pixel Accumulation Algorithm Based on KNN**
Input: Single photon matrix ***X***, Label matrix ***Y***, Ground truth matrix ***Z***
1:   **for** $z_{i,j}$ = ${\left[z_{i,j}^{1},z_{i,j}^{2},z_{i,j}^{3},\ldots ,z_{i,j}^{h}\right]}^{T}$ in ***Z*** **do**
2:      $f_{i,j}\;\underset{\leftarrow }{feature\;extraction}\;z_{i,j}$
3:   **end for**
4:   ***P*** = $\left\{(\right.$***f***_*i*,*j*_, *y*_*i*,*j*_)}
5:   **for** $x_{i,j}$ = ${\left[x_{i,j}^{1},x_{i,j}^{2},x_{i,j}^{3},\ldots ,x_{i,j}^{h}\right]}^{T}$ in ***X*** **do**
6:      $f_{i,j}^{\;x}$$\underset{\leftarrow }{feature\;extraction}$$x_{i,j}$
7:   **end for**
8:   ***B*** = $\left\{f_{i,j}^{x}\right\}$
9:   **for** $f_{i,j}^{x}$ in ***B*** **do**
10:      ***N***_*K*_$\left(f_{i,j}^{x}\right)$← K nearest points in ***P***
11:      $y_{i,j}^{\;x}$ = $\underset{c_{i,j}}{\arg\max}\sum_{p_{i,j}\in N_{K}\left(f_{i,j}^{x}\right)}\varphi \left(y_{i,j},c_{i,j}\right)$
12:   **end for**
13:   **for** $x_{i,j}$ = ${\left[x_{i,j}^{1},x_{i,j}^{2},x_{i,j}^{3},\ldots ,x_{i,j}^{h}\right]}^{T}$ in ***X*** **do**
14:      **if** $y_{i,j}^{\;x^{\prime }}$∈$N_{8}$($x_{i,j}$) = $y_{i,j}^{\;x}$ **then**
15:         $q_{i,j}$ = $x_{i,j}$ + $x_{i,j}^{\prime }$
16:      **end if**
17:   **end for**
Output: Data matrix ***Q***


**Figure 5:**
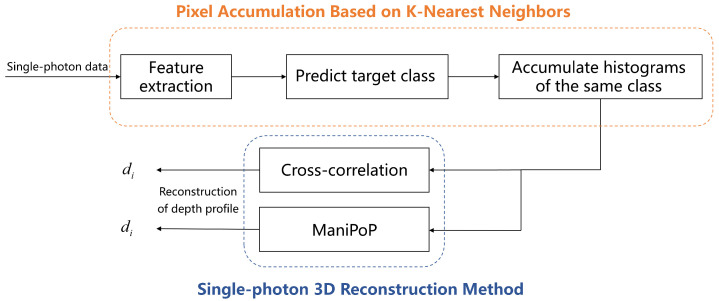
Diagram of KNN-based single-photon 3D reconstruction algorithm.

The single-photon data matrix after pixel accumulation is obtained through the proposed algorithm. Subsequently, the pixel cross-correlation algorithm and the ManiPoP algorithm are used to reconstruct the depth profile of the single-photon data matrix. The overall process of the KNN-based single-photon 3D reconstruction algorithm is shown in Figure 5.

## 4. Experimental Results and Analysis

The validation of the effectiveness of the proposed algorithm was divided into two parts: a real-world, land-based, single-photon data experiment and a simulated, underwater, single-photon data experiment. In the underwater simulated experiment, not only was the effectiveness of the proposed algorithm validated but, due to the parameter settings of the underwater simulation experiment being based on the SPAD underwater optical imaging experimental system parameters described in Section 2, the simulation experiment also served as a dual validation of the algorithm’s effectiveness and the feasibility of the designed SPAD imaging experimental system.

### 4.1. Single-Photon Experiment with Real Land-Based Target

In the validation of the effectiveness of the KNN-based single-photon 3D reconstruction algorithm, the data source was the single-photon dataset publicly released by Heriot-Watt University [21]. The experimental environment parameters for collecting single-photon data are shown in Table 2.

**Figure 6:**
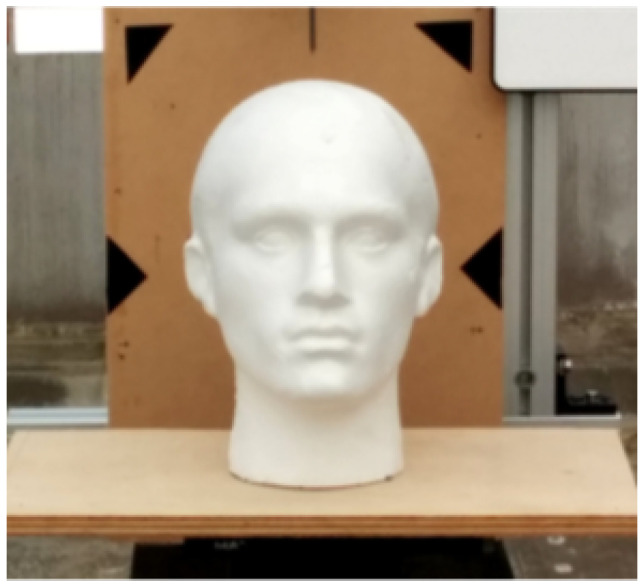
Image of the detection target.

The target was a polyethylene head model at 40 m. The image of the detection target is shown in Figure 6.

The spatial resolution of the single-photon data was 141 × 141 pixels, the number of time bins in the time–photon histogram was 4613, and the time resolution of the SPAD array was 2 ps. According to Equation (12), the depth resolution of the SPAD array was 0.3 mm.

(12) $$\Delta_{b}=c\frac{T_{b}}{2}$$

**Figure 7:**
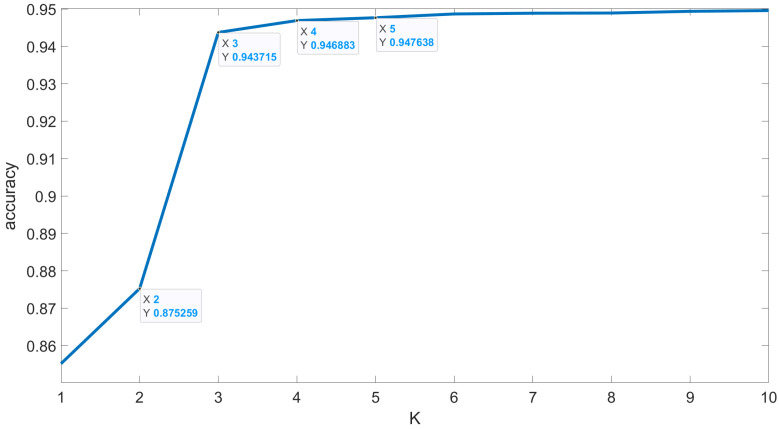
K-value cross-validation results of real land-based target.

Before the proposed algorithm was used to perform depth profile reconstruction, the optimal value of K in the KNN algorithm was determined through cross-validation methods, as shown in Figure 7. From the results, it can be seen that, when K changed from 2 to 3, the accuracy of KNN increased from 87.53% to 94.37%, an increase of 6.84%. The increase in accuracy when K was increased beyond 3 did not exceed 1%. Since the principle adopted during classification decision-making is “the majority rules”, the best value for K should be an odd number. Thus, in experiments with a land-based target, 3 was chosen as the value of K in the KNN algorithm.

After choosing the optimal K value through cross-validation, we applied various classification algorithms to classify the single-photon features of a land-based target. The specific results are shown in Table 3. According to the accuracy of classification results, KNN is not the best choice; however, while considering the running time of the algorithm, KNN is currently the most cost-effective choice.

**Figure 8:**
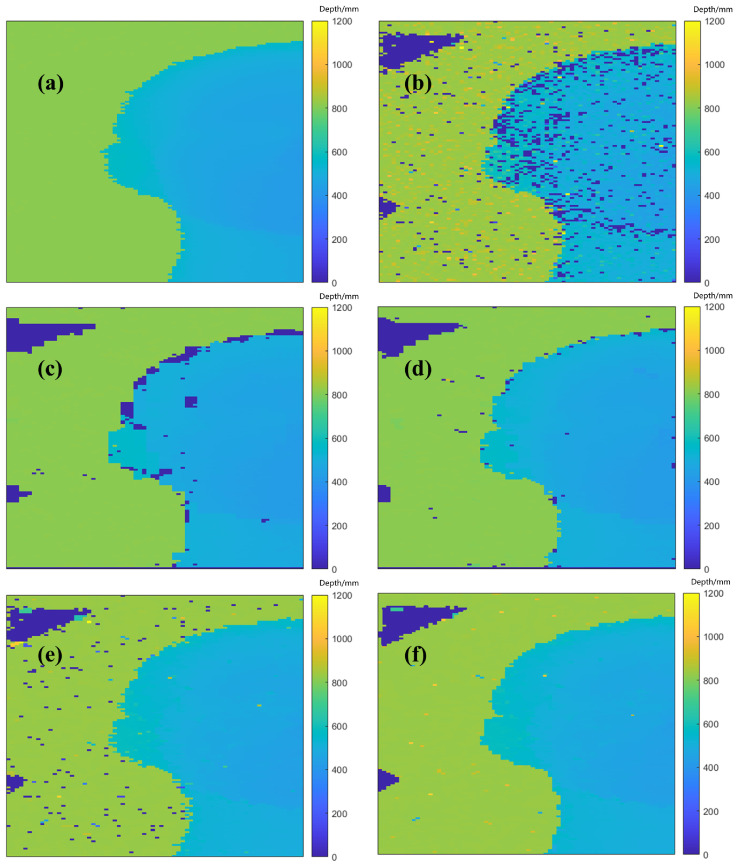
(**a**) Depth ground truth; (**b**) depth profile reconstructed via pixel cross-correlation algorithm; (**c**) depth profile reconstructed via the ManiPoP algorithm; (**d**) depth profile reconstructed via the ManiPoP algorithm based on the KNN algorithm; (**e**) depth profile reconstructed via the pixel cross-correlation algorithm after simple pixel accumulation; and (**f**) depth profile reconstructed via the pixel cross-correlation algorithm based on the KNN algorithm.

The reconstruction results of different algorithms are shown in Figure 8. In the results, the depth values are not the absolute depth measured via the detector but a relative depth. This means that an arbitrary zero point was chosen, and the depth was scaled to enhance the visual color effect.

As the visualization results show, in the depth profile of the original single-photon data reconstructed via the ManiPoP algorithm, most of the background noise was removed, but some noise was retained at the edges of the target head where it intersected with the background. However, in the depth profiles reconstructed from the single-photon histogram after processing with the pixel accumulation algorithm following the KNN algorithm, the edges of the target contour were clear, and the noise reduction effect between the background and the target was very evident. After simple pixel accumulation was performed on the original single-photon histogram and then the pixel cross-correlation algorithm was used to reconstruct the depth profile, the quality of the reconstructed result was not satisfactory, which was because simple pixel accumulation indiscriminately accumulates single-photon data, so the impact of noise may not be removed and can even be amplified. After the KNN algorithm was used for classification and then the pixel cross-correlation algorithm was used to reconstruct the depth profile, the noise in both the background and the target parts was significantly removed. Under conditions wherein the target’s millimeter-level high-resolution features could be observed, the denoising effect in the reconstructed depth profile was also significantly improved in the visualization results.

**Figure 9:**
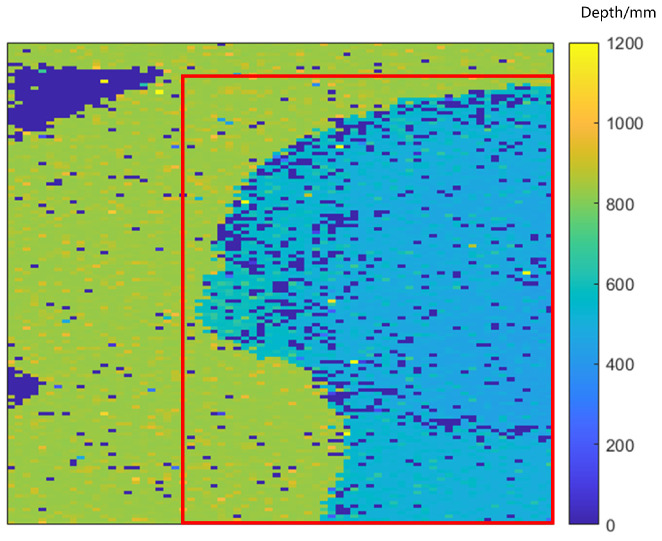
The area selected for error calculation.

To better evaluate the reconstruction results, the root mean squared error (RMSE) was used to quantify the advantages and disadvantages of the proposed algorithms compared to traditional 3D reconstruction algorithms. The calculation method is shown in Equation (13).

(13) $$RMSE=\sqrt{\frac{1}{n}\sum_{i=1}^{n}{\left(y_{i}-{\hat{y}}_{i}\right)}^{2}}$$

where $y_{i}$ represents the true depth of the target, ${\hat{y}}_{i}$ is the depth value of the target obtained through 3D reconstruction via the algorithm, and *n* represents the total number of pixels. Since the ground truth does not provide data for the black triangle on the background board in the actual experimental scene, to avoid the black triangle causing significant interference in the error calculation, the area within the red box shown in Figure 9 was selected for error calculation.

The RMSE of the real-world target depth profiles reconstructed via different algorithms is shown in Table 4.

The RMSE of the depth profile of a real-world target after pixel accumulation following the KNN algorithm, reconstructed with the ManiPoP algorithm, decreased by 26.83% compared to the original reconstruction results, which effectively improved the accuracy of the reconstructed depth profile. After simple pixel accumulation was performed on the original single-photon data and then the pixel cross-correlation algorithm was used to reconstruct the depth profile, the RMSE significantly decreased, indicating that simple pixel accumulation has a certain noise reduction effect on single-photon data. The result of the depth profile obtained via the pixel cross-correlation algorithm with the KNN algorithm showed an RMSE reduction of 59.96% compared to the original single-photon data reconstructed result, which represents a very significant improvement in reconstruction accuracy, aligning with the visual presentation effect.

In ideal conditions, such as long acquisition times, and without an environmental light influence, the depth resolution of the profile is limited only by the timing resolution of the system and the number of counts detected. In the real, land-based, single-photon dataset, the timing histogram bin width used was 2 ps, which allowed for the achievement of sub-millimeter depth resolution. In experiments for reconstructing real, land-based, single-photon data, the achievable reconstruction precision reaches the millimeter level, which is slightly higher than ideal conditions. This is due to the SPAD imaging system being more limited SPAD timing jitter in practice, preventing it from reaching detection precision under ideal conditions. Additionally, the performance of the reconstruction algorithm also affects the precision of reconstruction. Without being able to ignore SPAD timing jitter, accurate photon timestamps can be obtained through optical gating [11].

### 4.2. Underwater Simulation for Single-Photon Experiment

#### 4.2.1. Simulation Experimental Scheme

Due to the limitations imposed via the underwater sealed enclosure and cable manufacturing in an underwater SPAD optical imaging experiment system, conducting underwater imaging experiments is challenging. Therefore, we used underwater simulation experiment data to validate the feasibility of the designed system.

**Figure 10:**
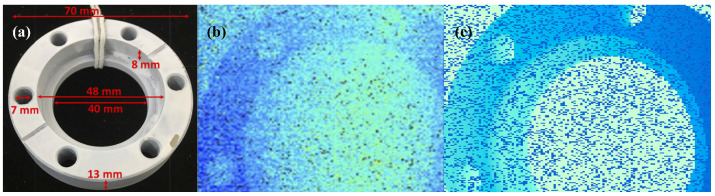
Comparison of simulated, underwater, single-photon, reconstructed depth profile and real, underwater, single-photon, reconstructed depth profile. (**a**) Simulation target; (**b**) depth profile reconstructed from real, underwater, single-photon data; and (**c**) depth profile reconstructed from simulated, underwater, single-photon data.

The underwater, single-photon imaging simulation included the absorption of light via water, with the forward and backward scattering of water particles in the medium. Figure 10 compares depth profiles reconstructed from underwater, simulated, single-photon data at 4.4 AL and real, underwater, single-photon data [19] at 4.4 AL using the cross-correlation algorithm. The result shows that the simulation of underwater absorption and scattering processes is feasible and convincing.

**Figure 11:**
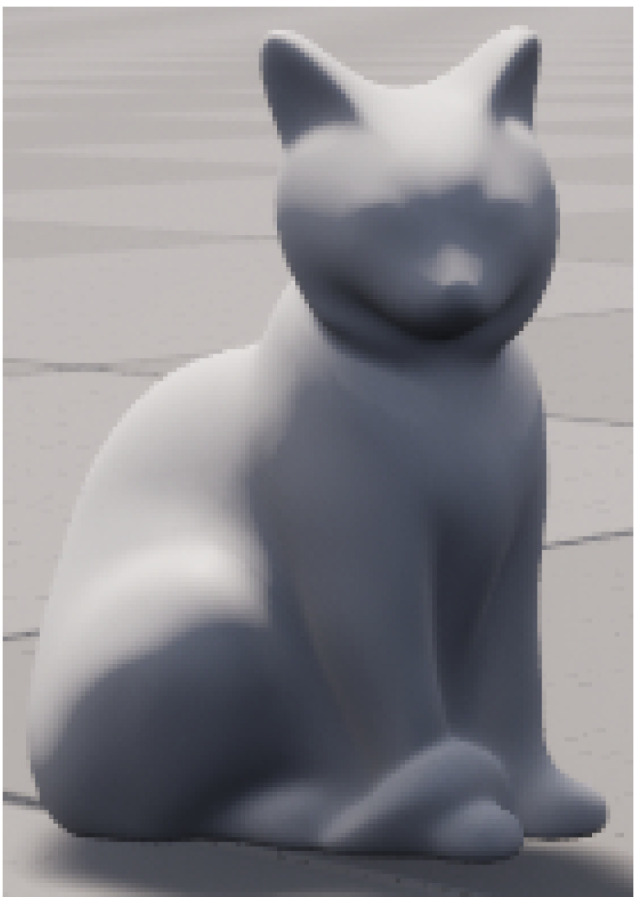
Image of simulation target.

In the virtual environment software Unreal Engine 5.0 developed by Epic Games, a three-dimensional target was constructed for the simulation experiment. The time–photon histogram of the target at a certain distance underwater was obtained [22]. The experimental depth profile was obtained through image processing algorithms and a 3D reconstruction algorithm, and the system’s feasibility and the proposed algorithm’s effectiveness were analyzed. The simulation target was an underwater sculpture of an animal at a distance of about 15 m and a vertical depth of about 1 m, as shown in Figure 11. The spatial resolution of the single-photon data for the simulation was 141 × 122 pixels, and the number of time bins in the TCSPC time–photon histogram was 200.

The simulation’s experimental parameters are shown in Table 5. The time resolution of PF32 SPAD was 55 ps, and according to Equation (12), the shortest resolvable distance was 16.5 mm, which enabled the differentiation of the simulation target. The attenuation coefficient of water for light is related to the wavelength of the light, suspended particles in the water, and other dissolved substances. Generally, the light attenuation coefficient in ocean waters is 0.1 m^−1^. In the simulation experiments, the absorption coefficient was set to 0.035 m^−1^, and the scattering coefficient was set to 0.4 m^−1^. Then, the light attenuation coefficient in the simulation experiment was 0.435 m^−1^. At this time, the effective distance corresponding to 15 m underwater was 6.5 AL.

**Figure 12:**
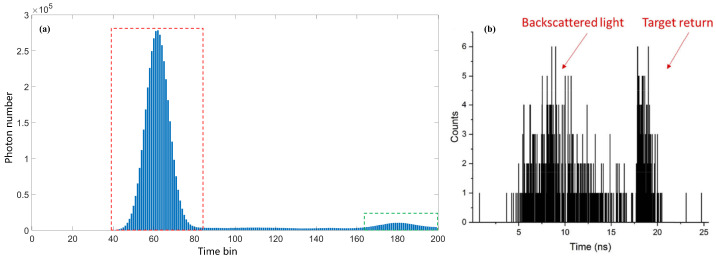
(**a**) Simulated underwater target echo signal; (**b**) real underwater target echo signal.

Figure 12 shows the time–photon histogram of the simulation target. The red-framed area represents the peak of scattering in the water, and the green-framed area represents the target peak. In this simulated time–photon histogram, the width of the time bin was 55 ps. The difference between the peak of scattering and the target peak was approximately 120 time bins, which was 6.6 ns. When comparing the simulated underwater time–photon histogram with the actual underwater time–photon histogram [19], it can be seen that the simulation results show a high similarity to the true results for both scattering and target echo signals, proving the feasibility of simulating underwater, single-photon detection experiments. Additionally, in the simulation results, the impact of scattering is much greater than its effect on target detection in the actual results, effectively reflecting the characteristics of high-scattering underwater environments.

#### 4.2.2. Simulation Experimental Results

**Figure 13:**
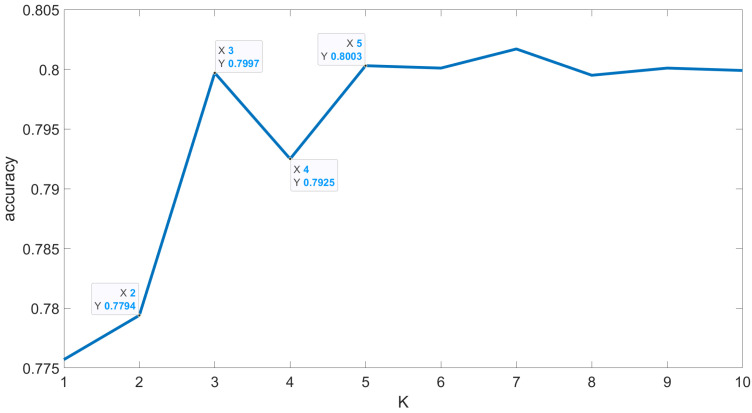
K-value cross-validation results of underwater simulation target.

Before the proposed algorithm was used to perform depth profile reconstruction, the optimal value of K in the KNN algorithm was determined through cross-validation methods, as shown in Figure 13. From the results, it can be observed that, when K changes from 2 to 3, the accuracy of the KNN algorithm improves by 2.03%. As K continues to increase, the predicted accuracy remains around 80%. Since the principle adopted during classification decision-making is “the majority rules”, the best value for K should be an odd number. Thus, in the underwater simulation, single-photon experiments, 5 was selected as the value of K in the K-nearest neighbors algorithm.

We also applied various classification algorithms to classify six different simulated underwater target single-photon features. The specific results are shown in Table 6. Compared with the land-based target, the classification accuracy of different algorithms for the simulated underwater target was a bit low. However, when considering the accuracy, with its corresponding uncertainty, and the running times of different algorithms, KNN is still the best choice.

**Figure 14:**
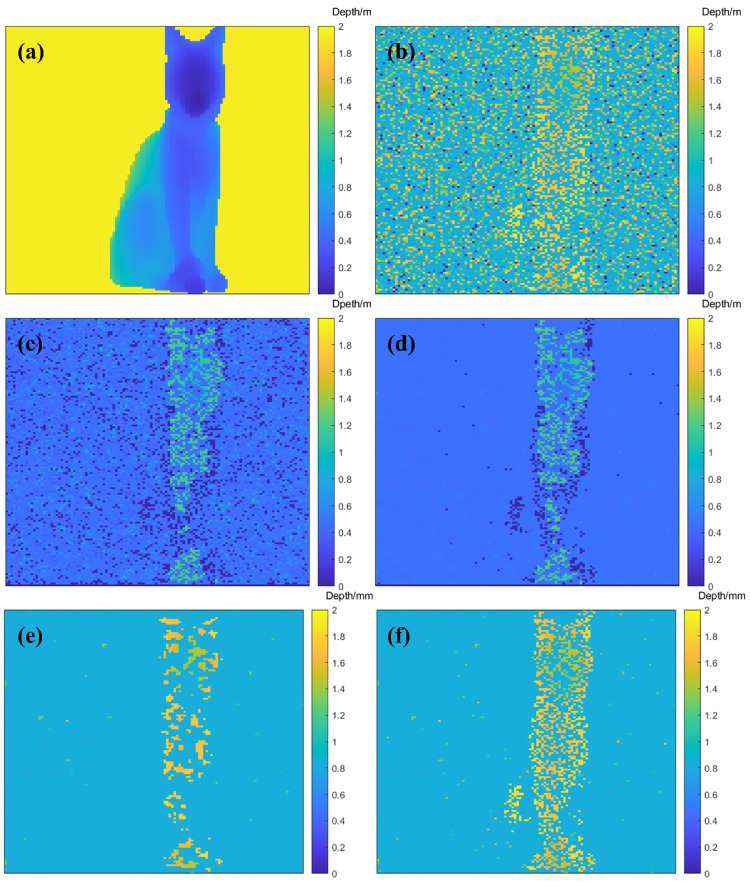
(**a**) Depth ground truth; (**b**) depth profile reconstructed via pixel cross-correlation algorithm; (**c**) depth profile reconstructed via ManiPoP algorithm; (**d**) depth profile reconstructed via ManiPoP algorithm based on KNN algorithm; (**e**) depth profile reconstructed via pixel cross-correlation algorithm after simple pixel accumulation; and (**f**) depth profile reconstructed via pixel cross-correlation algorithm based on KNN algorithm.

Different algorithms were used to reconstruct the simulated underwater single-photon data, as shown in Figure 14.

As the visualization results show, when the cross-correlation algorithm was used to reconstruct the depth profile, the noise reduction effect of simple pixel accumulation was evident, but the reconstructed target contour was not clear, and most of the depth estimates of the targets were not accurate. The SNR of the depth profile reconstructed from single-photon data after pixel accumulation based on the KNN algorithm significantly improved, and the reconstructed target was more complete, presenting a better visual effect. In the results of depth profile reconstructed via the ManiPoP algorithm, after pixel accumulation based on the KNN algorithm, most of the noise in the background was removed from the reconstructed results compared to the original reconstruction results, making the target easier to recognize.

To more clearly evaluate the advantages and disadvantages of different algorithms, the RMSEs of the reconstructed depth profiles were calculated. The RMSEs of the depth profiles reconstructed via different algorithms from six different simulated underwater single-photon data sets are shown in Table 7.

The RMSE of the depth profile of the simulated, underwater, single-photon data after pixel accumulation following the KNN algorithm, reconstructed via the ManiPoP algorithm, decreased by 6.29% compared to the original reconstruction results, which indicates an improvement in the accuracy of the reconstructed image. In the results of the depth profile reconstructed via the pixel cross-correlation algorithm, based on the reconstruction results processed with the proposed algorithms compared to the original reconstruction results, the RMSE decreased by 23.45%, which demonstrates a significant improvement in image reconstruction quality.

In experiments for reconstructing simulated, underwater, single-photon data, the achievable reconstruction precision was at the centimeter level. The main reason for this was that the backward scattering in underwater environments has a significant impact on imaging, affecting the estimation of background depth and resulting in final reconstruction depth precision at the centimeter level.

Based on the underwater 6.5 AL target reconstruction, simulations of single-photon detection were conducted for the same target at distances of 6.1 AL, 6.3 AL, 6.7 AL, and 7.0 AL, obtaining the corresponding underwater single-photon time histograms. The training data for the KNN algorithm were the ground truth of the 6.5 AL target time–photon histogram. The RMSEs of the reconstructed depth profiles via the proposed algorithm and others are shown in Table 8.

When the distance to the target changed, the training data for KNN were still the ground truth histogram of the target at 6.5 AL, and the RMSEs of the reconstruction results significantly decreased. After the ground truth histogram was obtained at a certain distance, the KNN-based single-photon 3D reconstruction algorithm still showed significant denoising effects for single-photon data at different distances, which demonstrates the superiority of the proposed algorithm in the denoising of underwater single-photon data. And the uncertainty corresponding to the RMSE also proves that the proposed algorithm has relatively high credibility in denoising underwater simulated single-photon data.

## 5. Conclusions

Due to the complex underwater imaging environment, the detection process is easily affected by water absorption and scattering, which leads to the low resolution of the target depth profile reconstructed from a time–photon histogram. Therefore, a single-photon histogram denoising algorithm is needed to reconstruct high-precision depth profiles from underwater single-photon data. It has been proven that simple pixel accumulation has denoising effects on single-photon histograms. However, it could blur the boundaries of the target and significantly increase the probability of depth estimation errors for targets. Therefore, the K-nearest neighbor algorithm was chosen to classify multiple targets and backgrounds in the same scene, thereby enhancing the noise reduction effect of the pixel accumulation algorithm and improving the quality of the reconstructed depth profile.

This paper designed an underwater SPAD optical imaging experimental system and conducted a simulation of the detection process for underwater target at 6.5 AL. Through simulation data, the feasibility of the SPAD underwater optical imaging experimental system and the effectiveness of the proposed KNN-based single-photon 3D reconstruction algorithm were validated. In the experiment with underwater simulated single-photon data, the RMSE of the depth profiles reconstructed from the time–photon histogram after processing with the pixel accumulation algorithm based on KNN decreased by 23.45% compared to the original time–photon histogram reconstruction results, which achieved centimeter-level reconstruction precision for underwater targets at 6.5 AL.

This paper also used a public dataset of single-photon data from real-world, land-based targets to validate the effectiveness of the proposed algorithm. When reconstructing depth profiles using the KNN-based single-photon 3D reconstruction algorithm, the reconstruction precision reached the millimeter level, and the error of the reconstruction results was reduced by 57.12% compared to the reconstruction results obtained using the pixel cross-correlation algorithm with the original single-photon data, which shows that the accuracy of the reconstructed depth profiles was significantly improved.

The main limitations of the proposed algorithm primarily stem from the KNN algorithm being a classic lazy learning algorithm, which does not have a training process but stores the training set in memory. As the SPAD array gradually increases in size, or when obtaining a large array of single-photon images through scanning, a considerable amount of memory space is required to store the data, which is the main constraint of the single-photon 3D reconstruction algorithm based on KNN. Future work can focus on ensuring algorithm effectiveness while reducing the amount of training set data, thereby lowering the demand for hardware platform performance and enhancing the applicability of the algorithm.

## Figures and Tables

**Table 1:** Summary of the main system parameters.

System Parameter	Value
Laser	Pulse semiconductor diode (PicoQuant VisUV-532)
Laser repetition rate	10 MHz
Illumination wavelength	532 nm
Average output power	100 mW
Laser pulse width	72 ps full width at half maximum
Image sensor	Photon Force PF32 SPAD array • 32 × 32 pixels • 20% (MLA) fill-factor • 50 μm pixel pitch • Up to 225k frames per second
Detection efficiency at λ = 532 nm	28%
TDC resolution	10 bit
TDC bin width	55 ps
SPAD jitter	150 ps full width at half maximum

**Table 2:** Experimental parameters.

System Parameter	Value	System Parameter	Value
Target size	170 × 285 × 250 mm	Wavelength	841 nm
Exposure time	30 ms	Laser repetition rate	19.5 MHz
Total scan time	10 min	Average power	240 μW

**Table 3:** Comparison of different classification algorithms in land-based data set.

Algorithm	Accuracy	Time
KNN	94.37%	4.157 ms
Random forest	93.89%	96.045 ms
Naive Bayes	95.17%	5.340 ms
AdaBoost	95.03%	220.755 ms
Discriminant analysis	94.91%	55.462 ms

**Table 4:** The RMSE of the real-world target depth profiles reconstructed via different algorithms.

Algorithm	Value
ManiPoP	120.63 mm
KNN-based ManiPoP	88.26 mm
Cross-correlation	180.42 mm
Cross-correlation after simple pixel accumulation	89.15 mm
KNN-based cross-correlation	72.24 mm

**Table 5:** Simulation experimental parameters.

System Parameter	Value	System Parameter	Value
Pulse energy	5 nJ	SPAD jitter	200 ps FWHM
Laser repetition rate	10 MHz	Focal length	50 mm
TDC resolution	55 ps	Aperture	F1.4
Illumination wavelength	532 nm	Attenuation length	6.5 AL
Laser pulse width	72 ps FWHM	Target reflectivity	99%
Exposure time	20 ns	Absorption coefficient	0.035 m^−1^
Detection efficiency at λ = 532 nm	28%	Scattering coefficient	0.4 m^−1^

**Table 6:** Comparison of different classification algorithms in simulated underwater data sets.

Algorithm	Accuracy	Uncertainty Quality	Time
KNN	80.61%	0.36%	4.298 ms
Random forest	77.84%	0.26%	120.354 ms
Naive Bayes	78.07%	0.07%	12.759 ms
AdaBoost	76.96%	0.55%	177.584 ms
Discriminant analysis	80.31%	0.73%	46.593 ms

**Table 7:** RMSEs of the depth profiles reconstructed via different algorithms from the simulated, underwater, single-photon data.

Algorithm	Value
ManiPoP	0.9343 ± 0.0231 m
KNN-based ManiPoP	0.8755 ± 0.0076 m
Cross-correlation	0.8584 ± 0.0160 m
Cross-correlation after simple pixel accumulation	0.7765 ± 0.0076 m
KNN-based cross-correlation	0.6571 ± 0.0045 m

**Table 8:** RMSEs of reconstructed depth profiles for underwater targets at different distances.

RMSE/m	Algorithm	Cross-Correlation	Cross-Correlation after Simple Pixel Accumulation	KNN-Based Cross-Correlation
Target Distance				
6.1 AL		0.9767 ± 0.0075	0.8285 ± 0.0023	**0.6985** ± **0.0031**
6.3 AL		0.9847 ± 0.0070	0.8451 ± 0.0030	**0.6830** ± **0.0029**
6.7 AL		0.9834 ± 0.0068	0.8556 ± 0.0021	**0.6965** ± **0.0035**
7.0 AL		0.9872 ± 0.0072	0.8402 ± 0.0032	**0.6779** ± **0.0032**

## Data Availability

This study’s dataset is available upon request from the authors.
